# OVARIAN CANCER IN OLDER ADULTS IN EAST INDIA

**DOI:** 10.1093/geroni/igae098.2314

**Published:** 2024-12-31

**Authors:** Esha Chakravarty

**Affiliations:** Calcutta Metropolitan Institute of Gerontology, Mitcham, England, United Kingdom

## Abstract

Ovarian cancer is a lethal gynaecological malignancy whose incidence rates increase with age and is called ‘silent killer’ due to lack of symptoms and late diagnosis. Cases of 500 elderly patients with ovarian cancer were studied with respect to age of diagnosis, survival rate and factors influencing survival from Calcutta Metropolitan Institute of Gerontology (CMIG), regional resource and training centre on ageing in East India. Fifty five percent of the patients had a diagnosis between 60-70 years, 25% had a diagnosis between 70-80 years and 20% at >80 years. The mean age of diagnosis was 69 years. The study found that cancer outcome deteriorates with age of the patient. The age standardized relative survival rates for 1 year was 57% for patients between 60-70 years, 43% for 70-80 years and 21% for >80 years. The 4 main factors for deceased survival in older women were found to be aggressive tumour and higher grade, resistance to chemotherapy, functional and cognitive decline and co morbid conditions. This study can help in introduction of decision aids that can will create a clear category of patients who will and will not benefit from cytoreductive surgery and chemotherapy and influence allocation of State resources.

